# Role of Follicle-Stimulating Hormone in Spermatogenesis

**DOI:** 10.3389/fendo.2018.00763

**Published:** 2018-12-14

**Authors:** Olayiwola O. Oduwole, Hellevi Peltoketo, Ilpo T. Huhtaniemi

**Affiliations:** ^1^Department of Surgery and Cancer, Institute of Reproductive and Developmental Biology, Imperial College London, London, United Kingdom; ^2^Cancer and Translational Medicine Research Unit, Laboratory of Cancer Genetics and Tumor Biology, Biocenter Oulu, University of Oulu, Oulu, Finland; ^3^Department of Physiology, University of Turku, Turku, Finland

**Keywords:** spermatogenesis, spermatogenic failure, gonadotropins, FSH, testosterone, sertoli cells, fertility

## Abstract

Spermatogenesis is a concerted sequence of events during maturation of spermatogonia into spermatozoa. The process involves differential gene-expression and cell-cell interplay regulated by the key endocrine stimuli, i.e., follicle-stimulating hormone (FSH) and luteinizing hormone (LH)-stimulated testosterone. FSH affects independently and in concert with testosterone, the proliferation, maturation and function of the supporting Sertoli cells that produce regulatory signals and nutrients for the maintenance of developing germ cells. Rodents are able to complete spermatogenesis without FSH stimulus, but its deficiency significantly decreases sperm quantity. Men carrying loss-of-function mutation in the gene encoding the ligand (*FSHB*) or its receptor (*FSHR)* present, respectively, with azoospermia or suppressed spermatogenesis. Recently, the importance of high intratesticular testosterone concentration for spermatogenesis has been questioned. It was established that it can be completed at minimal intratesticular concentration of the hormone. Furthermore, we recently demonstrated that very robust constitutive FSHR action can rescue spermatogenesis and fertility of mice even when the testosterone stimulus is completely blocked. The clinical relevance of these findings concerns a new strategy of high-dose FSH in treatment of spermatogenic failure.

## Introduction

Reproduction is controlled by the hormones functional in the hypothalamic-pituitary-gonadal (HPG) axis. In the male they concern the maintenance of testicular testosterone (T) production and spermatogenesis by the two pituitary gonadotropins, luteinizing hormone (LH) and follicle-stimulating hormone (FSH). The testicular target cells of LH are the Leydig cells present in the interstitial space, and those of FSH are the Sertoli cells present in the seminiferous tubules. LH stimulates Leydig cell T production, and FSH stimulates in Sertoli cells, in synergy with T, the production of regulatory molecules and nutrients needed for the maintenance of spermatogenesis. Hence, both T and FSH regulate spermatogenesis indirectly through Sertoli cells.

Although the principles of the hormonal regulation of spermatogenesis have been established decades ago, the recently acquired genetic information, in particular from human mutations of gonadotropin and gonadotropin receptor genes, and from genetically modified mouse models, have advanced our knowledge about the molecular events involved in the regulation of spermatogenesis. In this article, we review this information and describe some of our own studies on genetically modified mice, that reveal some new aspects of these regulatory events. Some of this information challenges the basic principles of the hormonal regulation of spermatogenesis, sheds light on its pathogenetic mechanisms, and offers new leads into its treatment.

## General Principles of Regulation of Spermatogenesis

Spermatogenesis is a complex and orderly sequence of events, during which diploid spermatogonia proliferate and differentiate into haploid spermatozoa in testicular seminiferous tubules (1, 2). In seminiferous tubules, the somatic Sertoli cells extend from the base of the tubule to its lumen and form a niche for germ cell maturation supporting the process qualitatively and quantitatively (1, 3–5). Sertoli cells also send signals, including paracrine factors and nutrients, to the germ cells. Starting at puberty, spermatogenesis normally continues uninterrupted throughout the lifespan (with seasonal variation in some animals) but decreasing somewhat in quantity with aging.

The spermatogenic process occurs in a stepwise fashion, and is regulated by the interplay of different autocrine, paracrine, and endocrine hormonal stimuli. The cascade involves a series of cellular mechanisms, which includes mitotic multiplication and propagation, meiotic recombination of genetic materials, and morphological maturation of spermatozoa (6, 7). The development and maintenance of spermatogenesis is dependent on the pituitary gonadotropins; FSH, and LH. Both hormones are secreted and regulated as a part of the HPG axis in response to the hypothalamic gonadotropin-releasing hormone (GnRH). GnRH stimulates the gonadotrophs in the anterior pituitary to secrete the gonadotropins in a pulsatile fashion into the systemic circulation. FSH and LH levels in turn are regulated by the negative feedback actions of gonadal sex steroids and inhibin that collectively downregulate GnRH secretion and maintain homeostasis of the HPG axis (8, 9).

FSH and LH mediate their individual actions on spermatogenesis through their cognate receptors, FSHR and LHR (LHCGR in humans). Both receptors are plasma-membrane associated G-protein coupled receptors, FSHR expressed on Sertoli cells and LHR on Leydig cells (10–13), where the latter stimulates T production (14, 15). T is considered a prerequisite for sperm production and maturation, secondary sexual characteristics and functions, and anabolic actions. T activates the androgen receptor (AR) in Sertoli cells to initiate the functional responses required for spermatogenesis (16). FSH, on the other hand, is considered to act both independently and in concert with T to stimulate Sertoli cell proliferation and to produce signaling molecules and nutrient to support spermatid maturation (11, 17).

## FSH is an Important Regulator of Sertoli Cell Proliferation

Sertoli cells form both structurally and biochemically a supporting environment for the maturing germ cells. Their number is determined by FSH action, in rodents during fetal and neonatal life, and in primates at neonatal and peri-pubertal age (5). In both rodents and primates, *FSHR* expression starts during the second half of gestation (18, 19), though the lack of ligand (FSH) and cAMP responsiveness imply that the receptor is initially functionally inactive (20). However, after the onset of fetal pituitary FSH production and activation of the receptor, the hormone plays a major role in Sertoli cell proliferation (21). During peri-puberty, the rising FSH concentration triggers the second phase of Sertoli cell proliferation (5, 21), and the concentration of circulating FSH correlates strongly with Sertoli cell number and testis size in adulthood (22, 23). In the absence of FSH or FSHR, the Sertoli cell number is considerably decreased, by 30–45%, in comparison to normal testicular development [Table 1,(5, 24, 32)]. This is of high importance, as the Sertoli cells number determines the quantity of sperm produced; a Sertoli cell is able to support a certain maximum number of germ cells (3, 5, 24, 26, 33, 34).

**Table 1:** Outcome of manipulation of FSH action and defects on testis mass, number of Sertoli cells, and completion of spermatogenesis.

**Treatment/mouse model/human condition**	**Species**	Testis mass or volume (%)^*^	Number of Sertoli cells (%)^*^	Number of round (r) or elongated (e) spermatids (%) or sperm count (s)^*^	Ratio of round spermatids to number of Sertoli cells (%)^*^	**Reference(s)**
*Fshb* knockout	Mouse	40	57–70	40(r), 37(e)	57	(24, 25)
*Fshr* knockout	Mouse	42	55^**^	36(r)^**^	69^**,***^	(26)
*FSHR* null mutation 566CT; A189V	Human	27–100	n/a	oligospermic-normospermic (s)	n/a	(27)
*FSHB* missense, frameshift and truncating mutations	Human	7–80	n/a or reduced number of Sertoli cells	azoospermic (s)	n/a	(28) and references therein
*Acvr2a* knockout (stimulation of FSH prevented)	Mouse	40–43	60–61	45(r), 41(e)	75	(24)
*hpg* (gonadotropin-deficient hypogonadal) + tgFSH expression	Mouse	500	162^**^	Increased, but low (r), hardly detectable (e)	Cannot be measured due to the absence of spermatids in *hpg* mice	(29)
*(hpg* + T implant) + tgFSH expression	Mouse	166	132^**^	161(r), 184(e)^**^	117^**^	(29)
Neonatal treatment with rhFSH	Rat	124	149	n/a	n/a	(30)
*Fshr*-CAMG1738C; D580HtgFSHR expression	Mouse	94	85	87(r), 87(e)	103	(31)
LuRKO; *Lhcgr* knockout	Mouse	19	29	4.8(r), 0(e)	16	(31)
*Fshr*-CAM/LuRKO crossbreed	Mouse	86	83	94(r), 51(e)	115	(31)

* *In comparison to corresponding controls in each experiment*.

** *Estimated from the charts presented in the article*.

*** *All germ cells/Sertoli cells; n/a, data not available; rh, recombinant human; tg, transgenic; CAM, constitutively activating mutation*.

## FSH Supports Spermatogenesis Quantitatively in Rodents

Classical studies on animal models indicate that Sertoli cells proliferate until a finite number and differentiate toward puberty. Prepuberty, together with increasing FSH secretion, *FSHR* expression begins to fluctuate along with the stage of spermatogenesis. This is associated with maturation of the Sertoli cell population and completion of the first cycle of sperm maturation (35). In the postpubertal testis, FSH together with T evokes in Sertoli cells signals to propagate germ cell maturation (5), to provide antiapoptotic survival factors and to regulate adhesion complexes between germ cells and Sertoli cells (36).

**Figure 1:**
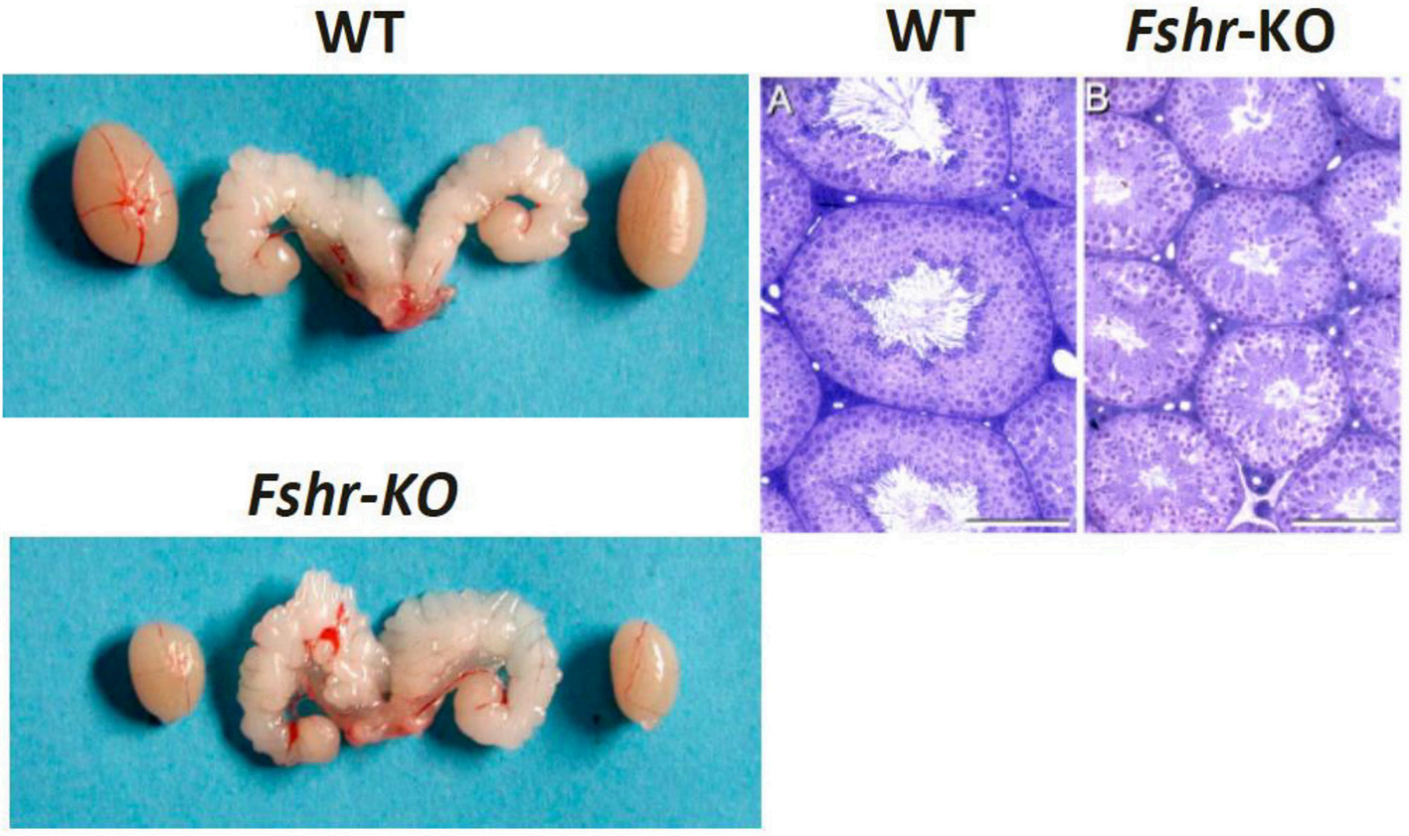
Testes and seminal vesicles of adult wild type (WT) and *Fshr*KO) mice **(Left)**, and testicular histology of same genotypes **(Right)**. No difference is observed in seminal vesicle sizes between the two genotypes, but the size of the *Fshr*KO testes is about half that of WT. Also, while full spermatogenesis is visible in the histology of both testes, the tubular diameter is clearly narrower in the knockout testis. From (37) with permission.

The lack of FSH (25) or FSHR (37, 38) in mice does not lead to sterility, albeit it decreased testis size (Figure 1), reflecting reduced Sertoli cell number and capacity to support and nurture germ cells (24, 26). *Fshb*- and *Fshr*-knockout (-KO) mice present with complete spermatogenesis, but the amount of germ cells remained lower than in control animals. In more detail, the KO mice have <50% of spermatogonial cells and ~50% of spermatocytes in comparison to wild-type animals, and the number of germ cells is reduced further to <40% of wild-type mice at postmeiotic stages [Table 1, (24, 26)]. While FSH influences solely the proliferation of Sertoli cells, T and FSH impact additively on the germ cells' entry into meiosis and stimulate synergistically its completion and entry into spermiogenesis (26). Experimental data from chemically and hormonally treated rats indicate that FSH is beneficial in the early stages of spermatogenesis until round spermatids, while the effect of T becomes enhanced thereafter (39–41). The germ cell to Sertoli cell ratio also decreases in the absence of FSH or FSHR [Table 1, (24, 26)]. Therefore, the reduction in the number of germ cells is not solely due to the decreased amount of the supporting Sertoli cells, but also because of their decreased ability to nurture germ cells.

## The Conundrum of the Role of FSH Deficiency in Human Spermatogenesis

To our knowledge, only one harmful (inactivating) *FSHR* mutation has been identified in men so far. In a cohort of several Finnish families of women with hypergonadotropic hypogonadism due to inactivating *FSHR-*A189V mutation, five male brothers were found to be homozygous carriers of the same mutation (27). While their homozygous female relatives had ovarian failure and infertility, the men had an unassuming phenotype which could not have been detected without the family connection. Four of the men were subfertile, phenocopying the FSH- and FSHR-deficient mice with complete but quantitatively reduced spermatogenesis, and two of them fathered two children each. In striking contrast, all men so far identified with *FSHB* mutation (*n* = 5) have been azoospermic and infertile (28). This was unanticipated, since receptor defects generally present with more severe effects in hormone action than those of ligands. An explanation for the discrepancy between the phenotypes of men with *FSHR* and *FSHB* mutations could be the residual activity of the mutant FSHR. Indeed, when highly overexpressed *in vitro*, a small fraction of the mutant FSHR-A189V is able to reach cell membrane, where it binds FSH and activates cAMP production (42). FSHR-A189V is also able to trigger mitogen-activated protein kinase phosphorylation via β-arrestins, thus activating the signaling cascade (43).

Additional genetic and/or environmental factors besides the *FSHB* mutations may also explain the azoospermia found in the mutation carriers. The *FSHB* mutations detected, however, are independent of each other, and the men are from different ethnic backgrounds supporting the essential role of FSH in human spermatogenesis. It would be expected that men with such mutation would respond to FSH treatment. One patient was found to attain a larger testis volume after 1-year treatment, but with spermatogenesis stalled at the spermatocyte stage (28). A moderate amount of missense, frameshift, and stop-gained mutations have been identified in *FSHB* and *FSHR* genes in ExAC database of over 60,000 individuals [http://exac.broadinstitute.org/gene/ENSG00000170820; http://exac.broadinstitute.org/gene/ENSG00000131808 (44)]. The constraint metrics for the gene variations suggest that they are well tolerated, and thus more homozygous mutation carriers may be identified in the future alongside increasing exome and whole genome sequencing. For now, with few men carrying known pathogenic *FSHB* or *FSHR* mutations, and other possible related factors affecting spermatogenesis unrevealed, it remains unclear whether FSH action is indispensable for spermatogenesis in men or playing a supporting role. One reason for the stark contrast between the numerous mutations detected in *LHCGR* and only few in *FSHR* may be the milder “real” phenotype of *FSHB/FSHR* mutations in men (45).

## Excessive FSH Action has Minor Influence on Testis Development and Function

There is a strong positive correlation between serum FSH concentration and testis development in rodents (22, 23). While the shortage of FSH or its receptor decrease spermatogenesis, neonatal administration of FSH increases to some extent the Sertoli cell number and testis size above normal in rats [Table 1, (30)]. Men with pituitary adenoma secreting excessive FSH appear to have normal testicular function (46, 47), suggesting that excessive FSH has no obvious effect in otherwise healthy men.

Gain-of-function (GoF) mutations of G-protein coupled receptor genes are rare. Few women with *FSHR* GoF mutations have been identified in pregnancy-associated ovarian hyperstimulation syndrome caused by constitutive activity and relaxed specificity of the receptor for hCG. However, the male relatives of the affected women did not have any reproductive or other health issues (48). A man with activating FSHR-D567G mutation has been identified to have normal spermatogenesis after hypophysectomy, even without T replacement therapy (49), suggesting that strong constitutive FSH stimulation can compensate for missing LH and reduced T action. In another case, a male carrier of an *FSHR*-N431I mutation was found to have complete spermatogenesis despite suppressed serum FSH (50). While the FSHR-D567G mutation increased basal cAMP production 1.5-fold *in vitro*, FSHR-N431I resulted in impaired agonist-stimulated receptor desensitization and internalization, thus causing “pseudo-constitutive” receptor activation. In both cases, the enhanced receptor activity likely compensated for shortage of the ligand. With no harmful effects on male health reported, there is the possibility that normal FSH activity brings about maximal physiological response in the male.

In general, recognition of GoF mutations should be easier than loss-of-function (LoF) mutations, because the former usually alter the phenotype in heterozygous form, while the LoF mutations must be homozygous (or compound heterozygous) to be effective. The scarcity of identified *FSHR* GoF mutations implies that they may not generally be harmful for their male carriers. This is in contrast to activating *LHR* gene (*LHCGR)* mutations that cause the dramatic phenotype of early-onset precocious puberty in boys (51, 52). The transgenic mouse line expressing *FSHR-*D580H in Sertoli cells supports the benign nature of GoF *FSHR* mutations in males (31). Despite robustly induced cAMP production in the absence of ligand (53) the transgenic males present with normal testis development and function, and do not differ significantly in Sertoli or germ cell number or fertility from their wild-type littermates [Table 1, (31)].

## FSH Regulates Genes Involved in Proliferation, Structure, and Function of Sertoli Cells

The molecular mechanisms of FSH action are discussed in other chapters of this special issue (Reiter and Casarini; Sayers and Hanyaloglu). We concentrate here on the processes and target genes of FSH action and their relationship to androgen action, to understand more precisely the role of FSH in spermatogenesis.

The regulatory system of the two gonadotropins, their feedback regulation, organization and interaction between germ and somatic cells, pose a challenge for dissecting the influence and target genes of a single factor, such as FSH. Sertoli cells present with a prominent fluctuating gene expression patterns along the seminiferous epithelial cycle (54). Therefore, their transcriptome profile analysis is highly dependent on sample source, time of collection, and culture conditions, as well as on the approach applied, *i.e*., microarray or transcriptome sequencing.

The general phenomenon is that FSH mostly elevates the expression of a large number of Sertoli cell genes (55–57). In neonatal life, Sertoli cells proliferate extensively, and logically mainly the transcripts of genes involved in DNA replication, cell cycle and stem cell factors are enriched (54). FSH most prominently stimulates many of these genes including *Krüppel-like factor 4, Klf4* (56, 58). KLF4 is a transcription factor that can be used to reprogram Sertoli cells to pluripotent stem cells (59), but it also plays a significant role in timing and accuracy of Sertoli cell differentiation (58). In GnRH-deficient hypogonadal (*hpg)* mice the proliferation and maturation of Sertoli cells are restrained (60), but their FSH stimulation triggers, as in neonatal mice, the expression of transcripts involved in RNA and DNA binding, cell cycle and cell growth, along with signal transduction and expression of transcription factors (56). The arrest of Sertoli cell proliferation alongside cell maturation is not only due to the cessation of proliferative gene expression, but also to upregulation of genes categorized in gene ontology as negative regulators of cell proliferation (54). While FSH-stimulated *hpg* mice presented with stimulation of proliferative factors and cessation of differentiating factors (56), chronically induced cAMP production by the FSHR-D567G mutation favored in cultured Sertoli cells the expression of genes involved in cellular differentiation at the expense of proliferation (61). One possible explanation for this difference could be biased signaling upon constant FSHR activation.

The mitotic quiescence of Sertoli cells is followed by formation of tight junctions and construction of the blood-testis barrier between mature Sertoli cells, in order to separate adluminal germ cells from the circulatory and lymphatic system (1). Androgens and FSH regulate in additive and synergistic fashion the expression of several genes adjusting blood-testis barrier dynamics or its components, including tight junction proteins and junctional adhesion molecules (54). Based on current evidence, androgen action is imperative for blood-testis barrier function. FSH, instead, has a more permissive role in stimulating the organization of inter-Sertoli junction types, and junctions between Sertoli cells and germ cells such as ectoplasmic specialization and adherent junction (56, 62, 63), thereby enabling the nurturing of germ cells. The Wnt pathway is one of those activated on postnatal days 5–10 in mouse Sertoli cells (54). FSH and T target a major morphogen, Wnt3, that in turn regulates the expression of *Gja*, which encodes a gap-junction protein essential for germ cell development (64).

Alongside Sertoli cell maturation, the transcript enrichment switches from proliferative and structural genes to those more involved in metabolic and germ cell supporting processes (54). Sertoli cell-produced retinoic acids are essential for the induction of spermatogonial differentiation during the first spermatogenic wave (65). Sertoli cells present with a specific temporally regulated array of genes related to retinoic acid synthesis and action (54), and FSH may affect both ligand metabolism and receptor function (66, 67). Logically for the supportive role of FSH in spermatogenesis, it also regulates and limits the massive wave of germ cell apoptosis during the first round of spermatogenesis (68–70). This process is apparently crucial to maintain the critical cell number between some germinal cell stages and Sertoli cells, and its lack brings about sterility (70). FSH also regulates the expression of genes involved in fatty acid metabolism and mitochondrial biogenesis, which is vital for the energy metabolism of seminiferous tubules (71). Another example of FSH-induced actions of Sertoli cells on germ cells is *Aqp8*, one of the most altered genes in the absence of FSH action (57). *Aqp8* is involved in water transport through membranes (72) and is strongly down-regulated in *Fshr*-KO mice (57). Expression of *Aqp8* as well as other FSH-stimulated genes is highly dependent on the hormone action during puberty in mice, but it returns to normal in adulthood even in the absence of the receptor, suggesting that FSH action on Sertoli cell function is not equally crucial after puberty (57). Finally, the partial and global gene expression profiles earlier referred to include numerous other less well characterized FSH-dependent genes. Their in-depth characterization will further elucidate the detailed mechanisms of FSH regulation of spermatogenesis.

## High Intratesticular T Concentration may not be Essential for Complete Spermatogenesis

FSH and T regulate several aspects of spermatogenesis independently, as well as in additive and synergistic manner (26). In contrast to FSH, there has been a consensus for the absolute requirement of T for spermatogenesis in most mammalian species. An exception is the photoperiod-dependent Djungarian hamster, where the restoration of spermatogenesis is dependent on FSH (73). In other mammals, the disruption of T production through hypophysectomy, Leydig cell ablation or knockout of *Lhcgr* results in interruption of spermatogenesis (74–77). The *AR* knockout mouse acts as a conclusive proof-of-concept that spermatogenesis will not proceed beyond meiosis without the support of T (78, 79).

The site of T production is the interstitial Leydig cells, and the local intratesticular concentration of T, depending on species, is in the order of 50–100-fold higher than that in systemic circulation (80–83). Studies in rodents indicate that intratesticular T below the normal high levels can support complete spermatogenesis. Both qualitatively and quantitatively normal spermatogenesis has been reported in rats at an intratesticular T concentration 30% of control animals (84). Full spermatogenesis has also been reported in T propionate-treated and hypophysectomized rats at intratesticular T concentrations below 5% of normal (85, 86). Furthermore, treatment of *hpg* (83, 87) and *Lhcgr* knockout (LuRKO) mice (88, 89) with subcutaneous T implants restored qualitatively complete spermatogenesis in a dose-dependent manner with minimal increase in intratesticular T concentration. Similarly, human carriers of partially inactivating *LHCGR* (90) and a LoF *LHB* mutation (91) have been reported to be oligozoospermic, instead of azoospermic, at very low serum and intratesticular T concentration. The evidence together indicates that high intratesticular T concentration is not a prerequisite for complete spermatogenesis, although it apparently increases sperm production and fertility.

## LH/T Regulation of Spermatogenesis can be Replaced by Strong FSHR Activation

Two recently used approaches have enhanced our knowledge about the role of FSH in spermatogenesis; the serendipitous discovery of mutations in human subjects and the generation of animals lacking or overexpressing FSH or its receptor. In clinical practice, patients presenting with hypogonadotropic hypogonadism are a valuable tool to study the role of gonadotropins in spermatogenesis and subsequent fertility. Those presenting with secondary hypogonadism consequent to idiopathic hypogonadotropic hypogonadism and Kallmann syndrome can be effectively treated with FSH and LH or with pulsatile GnRH administration (92–94).

**Figure 2:**
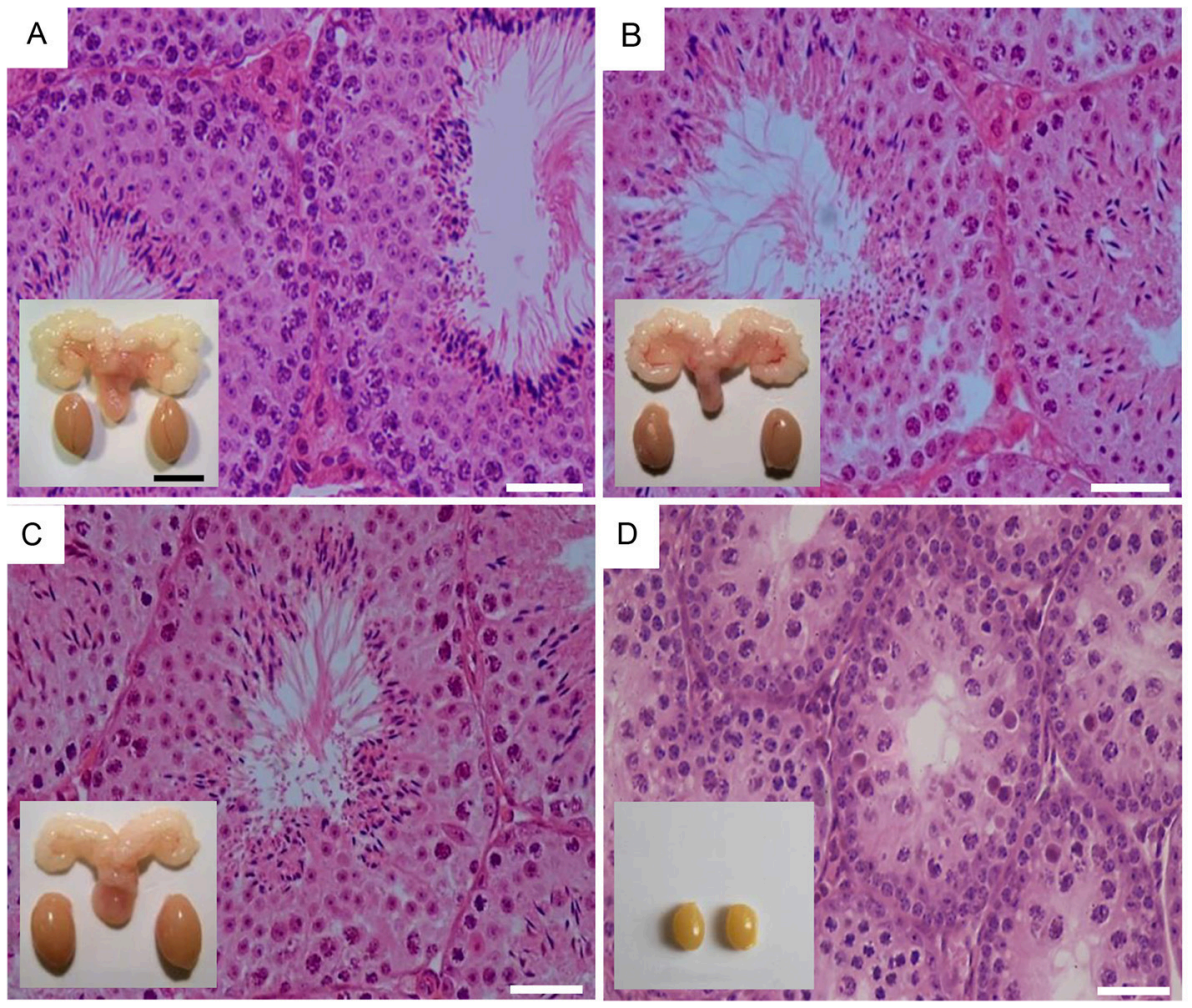
Testicular histology and macroscopic views of testes and urogenital blocks of different mouse genotypes: **(A)** WT, **(B)** *Fshr*-CAM, **(C)** *Fshr*-CAM/LuRKO, and **(D)** LuRKO mice. **(A–C)** show normal spermatogenesis and testis and seminal vesicle (SV) sizes. In **(D)**, spermatogenesis is arrested at the round spermatid (RS) stage, with small testes and rudimentary seminal vesicle (not visible). Scale bars: 50 μm; 10 mm (insets). From (31) with permission.

To dissect out the effect of excessive FSH action on spermatogenesis without simultaneous LH action, we took advantage of the transgenic mice expressing constitutively active *Fshr*-D580H (*Fshr*-CAM) driven by the anti-Müllerian hormone promoter in Sertoli cells (53). *In vitro*, FSHR-D580H induced without ligand cAMP production to the same degree as the wild-type receptor at saturating FSH concentration, thereby representing a strong constitutively active receptor. Female mice carrying the mutated gene developed several abnormalities in their ovaries and other estrogen target tissues, whereas the male littermates were devoid of any discernible phenotype deviating from normal (Figures 2A,B).

The *Fshr*-CAM mice (53) were then crossbred into the LuRKO (77) background to generate double-mutant *Fshr*-CAM/LuRKO mice with high FSHR signaling and minimal T production [Table 1, (31)]. Interestingly, the mutant *Fshr*-CAM expression reversed the azoospermia and partially restored fertility of the LuRKO mice to a near-normal male phenotype (Figures 2C,D, Table 1). Despite the absence of LHR in the double-mutant mice, intratesticular and serum T concentrations increased from the very low level in LuRKO mice to ~20 and 40% of those in wild-type mice. Anatomically, testicular and reproductive accessory gland development was normal (Figure 2C), and the mice were fertile, but presented with delayed puberty and small litter sizes compared to wild-type mice. The surprising fertility in these mice suggested that excessive FSH action could partially substitute for missing LH. A somewhat similar observation was made with the expression of *Fshr-*D567G in *hpg* background mice (29). In this transgenic model, the presence of the mutant *FSHR* also increased, to some extent, cAMP level in Sertoli cells and T production in absence of LH but was not able to rescue mature spermatogenesis. This indicates that very robust FSH action might be needed for successful spermatogenesis in the absence of sufficient T stimulation.

**Figure 3:**
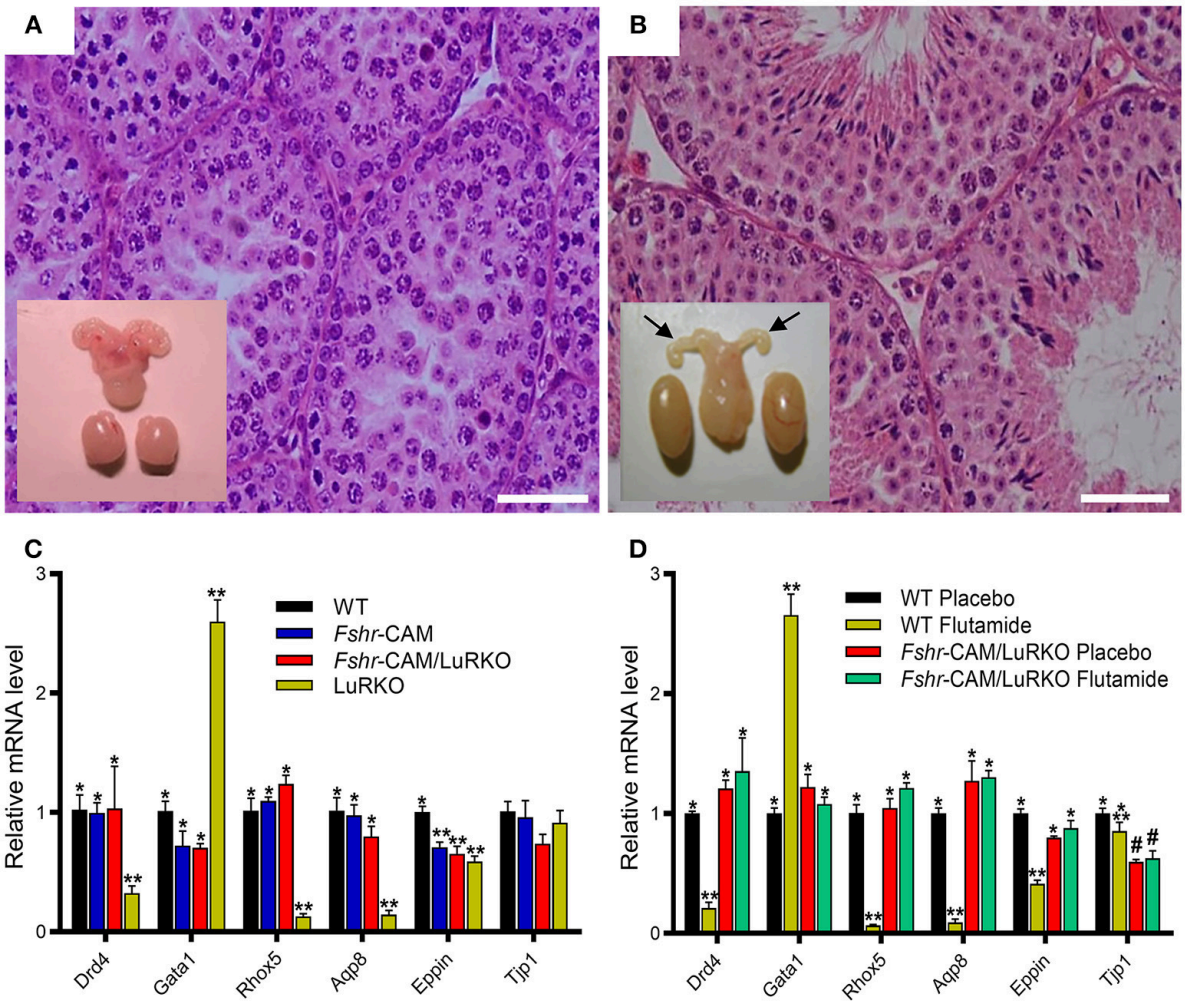
Effect of anti-androgen flutamide treatment on wild-type (WT) and genetically modified mice. **(A,B)** Testicular histology and macroscopic views of the testes and urogenital blocks of WT and *Fshr*-CAM/LuRKO mice. **(A)** The treatment arrested spermatogenesis at round spermatid stage in WT mice and reduced their testis and seminal vesicle sizes. **(B)** Identical treatment of *Fshr*-CAM/LuRKO mice had no apparent effect on their spermatogenesis and testis size but reduced seminal vesicle size (arrows in B). **(C,D)** Expression of selected target genes in untreated **(A)** and flutamide treated **(B)** mice. **(A)** Expression of androgen-regulated (*Drd5, Rhox5, Eppin*, and *Tjp1*), postmeiotic germ cell–specific (*Aqp8*), and germ cell–regulated (*Gata1*) genes in WT, *Fshr*-CAM, *Fshr*-CAM/LuRKO, and LuRKO testes. **(B)** Effect of flutamide treatment on expression of the same androgen-regulated genes in WT and *Fshr*-CAM/LuRKO mice. Data represent mean ± SEM. *n* = 3 samples/group. Bars with different symbols differ significantly from each other (*P* < 0.05; ANOVA/Newman-Keuls). The remarkable finding is that while flutamide treatment suppressed the expression of strictly androgen-dependent genes in WT mice, the same effect was not observed in the testis of *Fshr*-CAM/LuRKO mice. Scale bars: 50 μm. From (31) with permission.

To ascertain whether the observed residual Leydig cell T production was responsible for the spermatogenesis in *Fshr*-CAM/LuRKO mice, we completely blocked T action through treatment with the potent antiandrogen, flutamide. In wild-type mice, loss of T action by flutamide treatment led, as expected, to shrunken seminal vesicles and arrest of spermatogenesis at the round spermatid stage (Figure 3A). Unexpectedly, identical flutamide treatment of the *Fshr*-CAM/LuRKO mice prevented only the extragonadal androgen actions but had no deleterious effect on spermatogenesis (Figure 3B). This suggests that the constitutively active FSHR-D580H was able to maintain spermatogenesis, even after T action was completely abolished. Hence, the constitutively active FSHR-D580H astonishingly compensated for the action of the blocked LH/T pathway. Correspondingly, expression of several androgen-dependent Sertoli cell genes including *Drd4, Rhox 5, Aqp8, Eppin*, and *Gata1*, was decreased in the flutamide-treated wild-type mice as phenocopy of the LuRKO mice (Figure 3C), but the treatment had no effect on expression of these androgen target genes in *Fshr*-CAM/LuRKO mice (Figure 3D). The azoospermic phenotype observed in wild-type mice after androgen inactivation by flutamide was similar to that observed in Sertoli and peritubular myoid cell-specific *AR* knockouts mice (16, 79). The *AR* knockout mice demonstrate that spermatogenesis does not proceed to completion without the indirect T effect via Sertoli cells (78, 79). Therefore, the persistent spermatogenesis observed in the double mutant mice after flutamide treatment supports the conclusion that robust and constitutive FSHR activity can compensate for missing androgen action.

FSH and T have independent mechanisms of action; FSH acting through a membrane-bound G-protein coupled receptor, and T through AR, a nuclear transcription factor. However, when scrutinized in detail, overlapping mechanisms in their mode of action exist (95, 96). Both hormones activate the mitogen-activated protein kinase and cAMP-responsive element-binding protein signaling cascades, shown to be crucial for spermatogenesis through a rapid T signaling mechanism (97), and increase of Sertoli cell intracellular Ca^2+^ (98, 99). Thus, these partly overlapping mechanisms of androgen and FSH action may explain the ability of strong FSH action to substitute for the missing T stimulus. However, the quantitatively incomplete recovery of spermatogenesis in these mice emphasizes the importance of T for qualitatively and quantitatively full spermatogenesis.

## Summary and Clinical Implications

FSH function is an essential part of the complex HPG axis and its feedback control mechanisms in the regulation of testicular function. Pituitary-derived FSH provides indirect structural and metabolic support for development of spermatogonia into mature spermatids via its membrane-bound receptor in Sertoli cells. FSH also play a crucial role in determination of the number of Sertoli cells and thus their capacity to maintain spermatogenesis. In addition to proliferation and differentiation of Sertoli cells, FSH regulates the structural genes involved in the organization of cell-cell junctions as well as genes required for the metabolism and transport of regulatory and nutritive substances from Sertoli to germ cells. Although FSH is not a mandatory requirement for the completion of spermatogenesis in rodents, its deficiency, nevertheless, leads to significant reduction in sperm quantity. In humans, fertility phenotypes in carriers of inactivating *FSHB* or *FSHR* mutations varies from azoospermia to mild reduction of spermatogenesis.

In the past decades, men suffering from idiopathic hypogonadotropic hypogonadism have been treated with FSH in combination with LH to compensate for lack of endogenous gonadotropins (100). Due to the favorable influence of FSH on spermatogenesis, many studies of FSH administration have been conducted on men with idiopathic spermatogenic failure (101–103), but with variable outcome. The general conclusion from meta-analyses is encouraging on the effect of FSH on sperm quality (103), spontaneous pregnancy rates (101, 102), and pregnancies achieved through assisted reproductive techniques (102) in several, but not in all cases. FSH-treatment has improved not only the conventional sperm parameters such as motility, number and morphology of sperm, but also the non-conventional ones such as decreased amount of DNA damage and fragmentation (104, 105).

It is presumable that certain patient groups are more responsive to FSH treatment than others, depending on their genetic background and other factors involved. For example, individuals with hetero- or homozygous polymorphisms for serine in position 680 of the FSHR are shown to respond better to FSH treatment than those with asparagine in this position (106). On the other hand, FSH administration has been shown to decrease DNA fragmentation and thus improve the quality of DNA in patients with FSHR-N680, but not with FSHR-S680 (105). The dosage and length of FSH treatment also have marked effects on the outcome. Recent studies indicate that a sufficiently long FSH treatment, preferably at least 6 months, with a high dosage of at least 150 IU per injection every other day, can improve sperm parameters significantly more than the standard FSH treatments with lower doses (103). Ding et al. (107), showed convincingly an improvement of spermatogenesis and pregnancy rates in a group of idiopathic oligozoospermic men treated with increasing doses of FSH, with the best results achieved using administration of 300 IU of recombinant human FSH every other day for 5 months. Though results from mouse experiments cannot directly be extrapolated to humans, our recent studies with *Fshr*-CAM/LuRKO mice show that robust and constant FSHR stimulation can improve spermatogenesis and fertility rate even in the absence of T. However, based on the results from meta-analyses (101–103) caution is needed with the use of FSH, especially as it relates to high dosage and long-term treatments. In addition, more carefully controlled studies should be carried out to identify individuals with possible specific genetic makeup, who would most likely benefit from FSH treatment.

## Author Contributions

All authors listed have made a substantial, direct and intellectual contribution to the work, and approved it for publication.

### Conflict of Interest Statement

The authors declare that the research was conducted in the absence of any commercial or financial relationships that could be construed as a potential conflict of interest.

## Abbreviations

AR: androgen receptor
AQP8: aquaporin
CAM: constitutively activating mutation
DRD4: dopamine receptor D4
EPPIN: epididymal peptidase inhibitor
FSH: follicle-stimulating hormone
FSHβ: follicle-stimulating hormone subunit beta
FSHR: follicle-stimulating hormone receptor
GATA1: gata binding protein 1
GJA1: gap junction protein alpha 1 (Connexin43)
GnRH: gonadotropin releasing hormone
GoF: gain-of-function
hpg: hypogonadal
KLF4: krüppel-like factor 4
LH: luteinizing hormone
LHβ: luteinizing hormone subunit beta
LHR/LHCGR: luteinizing hormone/choriogonadotropin receptor
LoF: loss-of-function
RHOX5: reproductive homeobox 5
T: testosterone
TJP1: tight junction protein 1
WNT3: wnt family member 3
*Fshr-*CAM: constitutively active *Fshr*
*Fshr*-KO: *fshr* knockout
LuRKO: *lhcgr* knockout.
